# Single-Cell Analysis for Glycogen Localization and Metabolism in Cultured Astrocytes

**DOI:** 10.1007/s10571-019-00775-4

**Published:** 2019-12-20

**Authors:** Yuanyuan Zhu, Ze Fan, Rui Wang, Rougang Xie, Haiyun Guo, Ming Zhang, Baolin Guo, Tangna Sun, Haifeng Zhang, Lixia Zhuo, Yan Li, Shengxi Wu

**Affiliations:** 1 Department of Neurobiology, The School of Basic Medicine, The Fourth Military Medical University, Xi’an, China; 2 Department of Anesthesiology and Perioperative Medicine, Xijing Hospital of the Fourth Military Medical University, Xi’an, China; 3 Center for Brain Science, The First Affiliated Hospital of Xi’an Jiaotong University, Xi’an, China

**Keywords:** Astrocytes, Glycogen, Glycogen metabolism, Single-cell PCR, Fibrous astrocyte, Protoplasmic astrocyte

## Abstract

Cerebral glycogen is principally localized in astrocytes rather than in neurons. Glycogen metabolism has been implicated in higher brain functions, including learning and memory, yet the distribution patterns of glycogen in different types of astrocytes have not been fully described. Here, we applied a method based on the incorporation of 2-NBDG, a d-glucose fluorescent derivative that can trace glycogen, to investigate glycogen’s distribution in the brain. We identified two types of astrocytes, namely, 2-NBDG^I^ (glycogen-deficient) and 2-NBDG^II^ (glycogen-rich) cells. Whole-cell patch-clamp and fluorescence-activated cell sorting (FACS) were used to separate 2-NBDG^II^ astrocytes from 2-NBDG^I^ astrocytes. The expression levels of glycogen metabolic enzymes were analyzed in 2-NBDG^I^ and 2-NBDG^II^ astrocytes. We found unique glycogen metabolic patterns between 2-NBDG^I^ and 2-NBDG^II^ astrocytes. We also observed that 2-NBDG^II^ astrocytes were mainly identified as fibrous astrocytes but not protoplasmic astrocytes. Our data reveal cell type-dependent glycogen distribution and metabolism patterns, suggesting diverse functions of these different astrocytes.

**Electronic supplementary material:**

The online version of this article (10.1007/s10571-019-00775-4) contains supplementary material, which is available to authorized users.

## Introduction

Glial cells are the main type of neural cell and exist throughout the central nervous system (CNS) (Gallo and Deneen 2014; Brosius Lutz and Barres 2014; Walsh et al. 2014). Estimates regarding the ratio of glial cells to neurons vary greatly. However, the number of glial cells likely is at least equal to or exceeds the number of neurons. Among glial cells in mammalian brains, 20–40% are specifically defined as astrocytes, although the percentage of astrocytes has considerable variability across species and brain areas (Khakh and Sofroniew 2015). Astrocytes play important roles in the CNS, including roles in brain development, synaptic plasticity, synaptic transmission, blood flow regulation, energy metabolism, blood–brain barrier formation, circadian rhythm regulation, lipid metabolism, and neurogenesis (Guillamon-Vivancos et al. 2015; Lanciotti et al. 2013).

Brain glycogen is principally localized in astrocytes rather than in neurons (Magistretti and Allaman 2018; Gotoh et al. 2017). Astrocytes provide a rapid fuel supply for neighboring neurons through glycogenolysis, which is essential for learning and memory (Muller et al. 2014; Lalo et al. 2014). The brain glycogen stored in astrocytes was reported to activate the neuronal system, and the level of astrocytic glycogen increased during anesthesia and sleep (Brown and Ransom 2015; Zhang et al. 2016). Although astrocytes are strongly heterogeneous, including their morphology and function (Sun et al. 2010; Lukaszevicz et al. 2002; Miller and Raff 1984), the differences in glycogen distribution and metabolism among various astrocytes are unclear.

The green fluorescent d-glucose derivative 2-NBDG was developed by Yoshioka et al. (1996). Glycogen can bind with 2-NBDG, allowing the level of glycogen to be detected by quantifying the fluorescence intensity (Louzao et al. 2008). Therefore, 2-NBDG is a useful tool for studying glycogen in vivo and in vitro. Here, we applied 2-NBDG to observe the brain glycogen distribution in different astrocytes. Instruments and technologies enabling the isolation of individual single cells are required to deeply understand the natural properties of cells (Stumpf et al. 2015). To explore whether glycogen metabolism patterns are associated with the diversity of astrocytes, we developed a multidisciplinary approach to investigate the expression levels of glycogen metabolic key enzymes in single astrocytes. Whole-cell patch-clamp and fluorescence-activated cell sorting (FACS) were used to explore the astrocytic glycogen distribution.

In this study, we roughly divided astrocytes into two cell types based on glycogen localization and metabolism. Thus, our research seeks to characterize differences in astrocyte glycogen metabolism according to cell type heterogeneity, deduce possible functional differences, and contribute to knowledge on brain glycogen.

## Methods

### Primary Astrocyte Culture

Primary astrocytes were prepared from the brain cortex of C57BL/6 day 1 or day 2 newborn mice from the Fourth Military Medical University Laboratory Animal Center (Xi’an, China). The experimental protocols were reviewed and approved by the Ethics Committee of the Fourth Military Medical University. Briefly, the brain cortex was cut into small pieces followed by trypsin-EDTA (Gibco) treatment for 10 min. The tissue dissociated with trypsin was inactivated in Dulbecco’s modified Eagle’s medium (DMEM) (Gibco) and 10% heat-inactivated fetal bovine serum (FBS) (Gibco) and filtered through a 70-µm nylon cell strainer (BD Falcon). The filtered cells were plated on poly-d-lysine (PDL)-coated (sigma) culture dishes at a density of ~ 1 × 10^5^/cm^2^. Astrocytes were maintained in culture in DMEM, 10% heat-inactivated FBS, 1% penicillin & streptomycin Pen Strep (Gibco), and 1% glutamine (Sigma). After 1 week, the cells were shaken overnight for 19 h to remove nonspecific glia.

### Immunoblotting

Cultured astrocytes were lysed in RIPA buffer with 13 complete protease inhibitors (Roche). Protein levels were assessed with a Bradford assay with BSA as the standard. Approximately 10 µg of denatured proteins was separated by 8% SDS-polyacrylamide gel electrophoresis and blotted onto 0.22 µm PVDF membranes (Roche). Nonspecific binding was blocked with TBST (TBS-0.1% Tween-20) with 3% (w/v) nonfat milk for 2 h at room temperature. Membranes were incubated overnight at 4℃ in TBST with 5% milk and the following primary antibodies: rabbit anti-GFAP (1:1000, 20,044,021, Dako), rabbit anti-MAP2 (1:500, 17,490–1-AP, Proteintech), rabbit anti-S100 beta (1:1000, ab52642, Abcam), and mouse anti-β-actin (1:5000, 60,008–1-IG, Proteintech). Membranes were then incubated at room temperature for 2 h in TBST with 5% milk and secondary antibodies (1:5000, Invitrogen). Protein bands were detected by chemiluminescence (Tanon, Shanghai, China) and quantified by densitometry with ImageJ (ImageJ 7.0 software). Protein levels were normalized to the level of β-actin as a control.

### Electron Microscopy Analysis

Mouse cortical astrocytes were fixed in 4.0% glutaraldehyde for 2 h at room temperature on a shaker. They were rinsed in PB buffer, gently scraped, and postfixed in 1.0% osmium tetroxide in cacodylate buffer for 2 h on ice. The astrocytes were then rinsed again in PB buffer and dehydrated through an ethanol gradient of 30% to 100%. They were infiltrated with Epon 812 resin in a 1:1 solution of Epon:acetone soak for 45 min at room temperature. Then, they were placed in fresh Epon for hours and embedded in Epon overnight at 60 °C. These sections were cut on an ultramicrotome (Leica, Germany), collected on formvar-coated grids, stained with uranyl acetate and lead citrate, and examined using an electron microscope (Japanese electronics, Tokyo, Japan) at 80 kV. Images were collected using an AMT digital imaging system.

### Fluorescent Glycogen Detection with 2-NBDG in Cultured Astrocytes

Primary astrocytes were seeded on slides in 24-well plates at a density of 1 × 10^5^/well. When the cell confluence reached 80%, astrocytes were washed three times with PBS. Then, astrocytes were incubated at 37 °C with 2-NBDG (Cayman) at different concentration or time intervals. At the end of incubation, astrocytes were washed three times with PBS to wash out uncombined 2-NBDG. The slides with cells were removed from the 24-well plates. Then, the retained fluorescence was measured by a confocal microscope (Olympus, Japan) fluorescence reader at 488 nm excitation and 500–530 nm emission wavelengths.

### Fluorescent Glycogen Detection in a Microplate System

We used cells 3 or 4 days after seeding in 96-well plates, when confluence was reached. First, astrocytes were washed three times with PBS. Astrocytes were then incubated with 2-NBDG at 37 °C. At the end of incubation, astrocytes were washed three times with PBS. Then, astrocytes were incubated with 0, 1, 5, or 10 µM insulin (I9278, Sigma) and 10 µM adrenaline (A0937, Sigma) for 30 min. Next, astrocytes were again washed three times with PBS. Then, the retained fluorescence was measured by using an M200 PRO (infinite/TECAN, Switzerland) microplate fluorescence reader at 488 nm excitation and 500–530 nm emission wavelengths.

### Single-Cell qRT-PCR Analysis

Single astrocytes with different fluorescence signals were observed under a confocal microscope, and green fluorescence-positive cells were identified in photographs taken by a digital CCD. A single positive cell or negative cell was aspirated by a patch clamp under a Cell Selection and Transfer System attached to the fluorescence microscope. Each single cell was ejected into a centrifuge tube with 5 µL of DNase and RNase-free water. The total RNA from single astrocytes was isolated using a REPLI-g® WTA Single Cell kit (Qiagen) according to the manufacturer’s instructions. The mixture was incubated at 95 °C for 5 min and then cooled. Target cDNA levels were determined by RT-PCR (Thermo Fisher, Wilmington, USA) using SYBR Green (TaKaRa). Amplification assays were performed in 25 µL reaction mixtures containing TB Green Premix. PCR was performed using 2 µL of the cDNA solution, 12.5 µL of TB Green Premix, 1 µL of each primer (10 µM), and 8.5 µL of water. The PCR profile was 1 min at 95℃, 45 cycles of 5 s at 95℃, and 20 s at 60℃. The cDNA was normalized with SYBR qRT-PCR primers for mouse GAPDH. The forward and reverse PCR primers of glycogen synthesis-associated enzymes were as follows: glycogen synthase 1 (GYS1): 5′-TCAGAGCAAAGCACGAATCCAG-3′ and 5′-CATAGCGGCCAGCGATAAAGA-3′; glycogen synthase 2 (GYS2): 5′-ATCCCATCCTCAGCACCATTAGA-3′ and 5′-AAGGTGACAACCTCGGACAAACTC-3′; glycogen branching enzyme (GBE1): 5′-ACTACCGAGTCGGGACAGCAA-3′ and 5′-GGTCCAGTCTCTGATGACCTCCATA-3′. The forward and reverse PCR primers of glycogen breakdown-associated enzymes were as follows: brain type glycogen phosphorylase (PYGB): 5′-GCAGACTATGAAGCCTACATCCA-3′ and 5′-AGAACTTGCCAGAGCAGGCTATATT-3′; muscle type glycogen phosphorylase (PYGM): 5′-TCAACTGCCTGCACATCATCAC-3′ and 5′-CATGATAGTCCTCGGCACCATAAAC-3′; liver type glycogen phosphorylase (PYGL): 5′-ACCTCTGTGGCAGAAGTGGTGA-3′ and 5′-CCGATAGGTCTGTGGCTGGAA-3′; glycogen debranching enzyme (AGL): 5′-ACTGTGGCACGTGGATGGATAA-3′ and 5′-CCCACGATTTCCACAGCAGA-3’.

### Fluorescence-Activated Cell Sorting (FACS)

Primary astrocytes were seeded on the crawl after 2 or 3 days, when confluence reached 80%. First, astrocytes were washed three times with PBS. Then, astrocytes were incubated at 37 °C with 2-NBDG. At the end of incubation, astrocytes were washed three times with PBS. The astrocytes dissociated with trypsin were inactivated in DMEM and 10% FBS and washed three times with PBS. For each sample, 100 μL of astrocytes solution was added to 400 μL of PBS containing 1% FBS, as this relationship was found to be optimal for sample acquisition and analysis. All flow cytometry samples were prepared in round-bottom polypropylene FACS tubes (Falcon). After astrocytes were filtered through a 70-µm nylon cell strainer, the cells were analyzed by using a Sony SH800 cell sorter and a flow cytometer equipped with a 488-nm laser (Sony Biotechnology, Japan). Ten thousand events were collected using a forward scatter threshold of 50,000 (5%). Data on the pulse height, area, and width parameters were collected from the FITC fluorescence channel. Dead cells and debris were excluded by the FSC/SSC (forward scatter/side scatter) dot plot. All flow cytometry data were analyzed with FlowJo software (TreeStar, USA).

### Immunofluorescence

The astrocytes with 2-NBDG were gently rinsed with 0.01 mM phosphate buffer (pH 7.4) and then fixed for 5 min with 4% paraformaldehyde. The astrocytes were incubated overnight with 0.01 mM PBS (pH 7.4) containing 0.3% (v/v) Triton X-100 and 3% (v/v) bovine serum albumin (BSA) with a mixture of rabbit anti-GFAP (1:300, 20,044,021, Dako), rabbit anti-S100 beta (1:100, ab52642, Abcam), rabbit anti-MAP2 (1:300, 17,490–1-AP, Proteintech), goat anti-Iba1 antibody (1:300, AB5076, Abcam), rabbit anti-Oligo2 antibody (1:500, AB9610, Millipore), or mouse anti-A2B5 antibody (1:1000, ab53521, Abcam). The astrocytes were rinsed with PBS and incubated for 2 h in PBS with Alexa 647-AffiniPure Donkey anti-mouse IgG antibody (1:1000, 131,725, Jackson), Alexa Fluor™ 594 donkey anti-rabbit IgG antibody (1:1000, 1,890,862, Invitrogen), Alexa Fluor® 594 donkey anti-goat IgG (1:1000, 1,445,994, Life Technologies), and fluorescein (FITC)–conjugated AffiniPure goat anti-chicken IgG (1:1000, 143,125, Jackson). The astrocytes were mounted onto gelatinized glass slides and coverslipped with 50% (v/v) glycerol. The astrocytes were observed under a confocal microscope with appropriate laser scanning and filters for Alexa 488, Alexa 594, and Alexa 647. We performed control experiments in which the primary antibody was used. No labeling was observed under these conditions.

### Statistical Analysis

Data are presented as the mean ± SEM. Statistical significance was evaluated using Student’s *t* test or one-way ANOVA followed by the Tukey–Kramer post hoc test or the Dunnett post hoc test (GraphPad Prism 7.0 software). *p* < 0.05 was used to determine significance where indicated.

## Results

### Heterogeneous Glycogen Distribution in Cultured Astrocytes

**Fig. 1:**
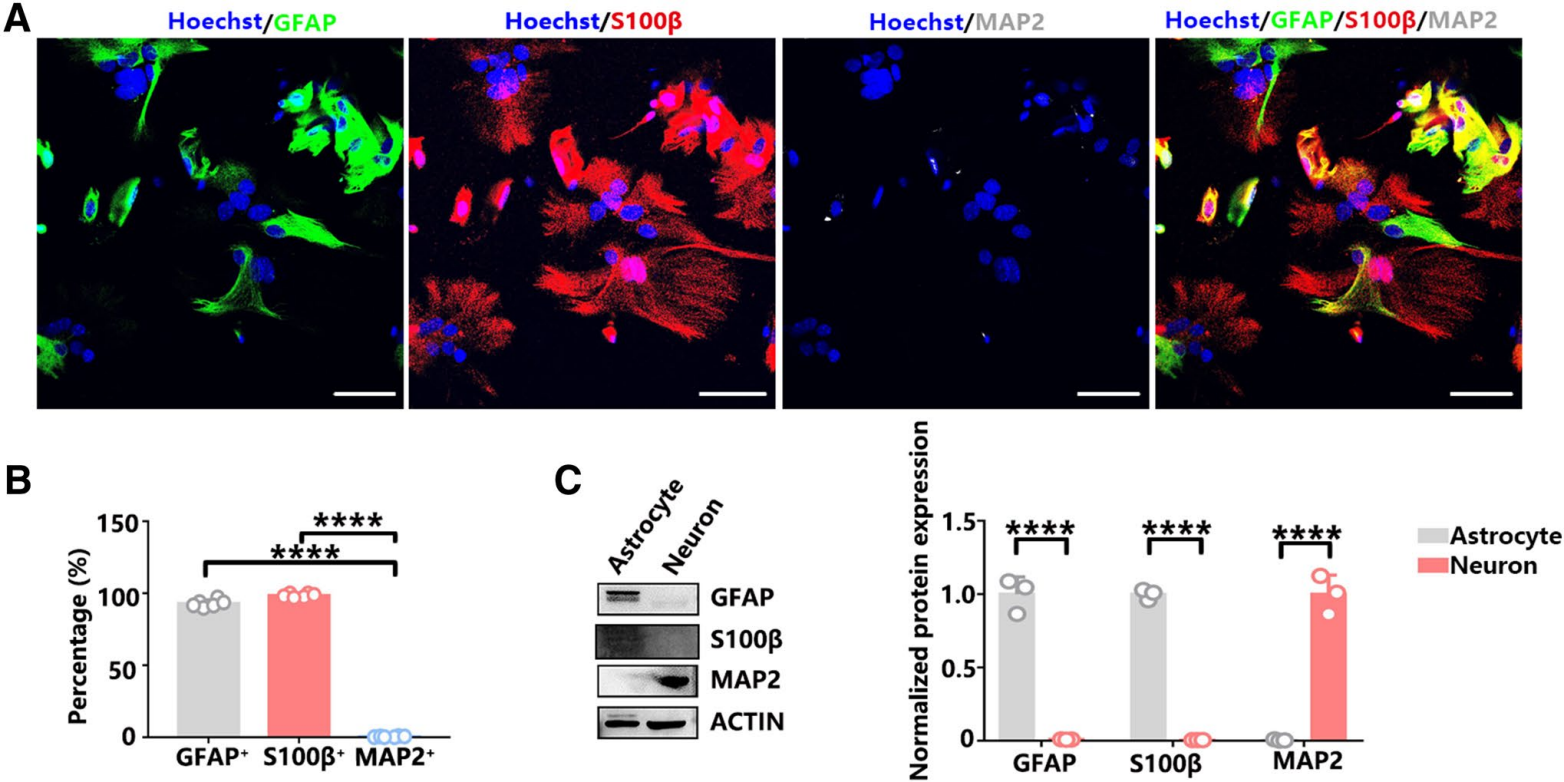
The purity of the astrocytes was confirmed by immunofluorescence and immunoblotting. **a** Costaining with GFAP (green), S100β (red), and MAP2 (gray, pseudo-color) confirmed the purity of the astrocytes. Scale bars = 50 µm. **b** Percentage of GFAP-, S100β-, and MAP2**-**positive cells. Statistical significance was evaluated using one-way ANOVA followed by the Tukey–Kramer post hoc test. *N* = 6 biological replicates. ^****^p < 0.0001. **c** Immunoblotting confirmed that the cultured cells in vitro were pure astrocytes. Statistical significance was evaluated using Student’s *t* test, *N* = 3 biological replicates. ^****^p < 0.0001

**Fig. 2:**
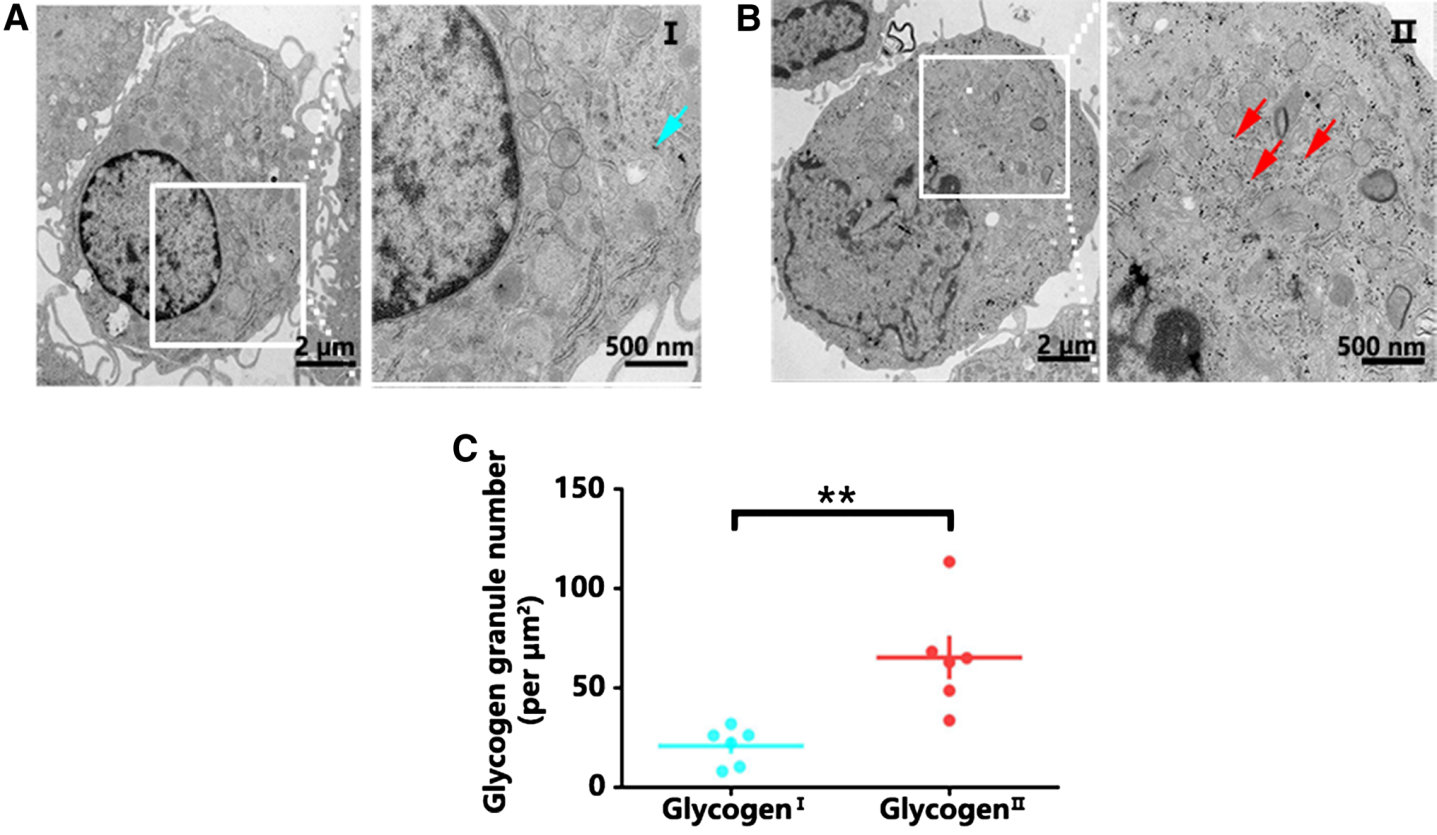
Distribution of glycogen in different types of astrocytes by electron microscopy. **a** Scanning electron microscopy photograph of glycogen^I^ astrocytes. Blue arrows indicate glycogen granules in glycogen^I^ astrocytes, scale bar = 2 µm, and a partially enlarged figure, scale bar = 500 nm. **b** Scanning electron microscopy photograph of glycogen^II^ astrocytes. Red arrows indicate glycogen granules in glycogen^II^ astrocytes, scale bar = 2 µm, and a partially enlarged figure, scale bar = 500 nm. **c** The glycogen content in glycogen^II^ astrocytes was significantly higher than that in glycogen^I^ astrocytes. Statistical significance was evaluated using Student’s *t* test, *N* = 6 biological replicates. ^**^p < 0.01

Fibrous and protoplasmic astrocytes are distinct types of astrocytes that differ in their antigenic phenotype and developmental history, as well as in their morphology and location within the CNS (Miller and Raff 1984). In fact, astrocytes exhibit substantially different ultrastructure under transmission electron microscopy (Castejon 2013). Therefore, we first observed the glycogen distribution in separated mouse brain cortex astrocytes. The purity of the cultured astrocytes was confirmed by immunofluorescence and immunoblotting analysis with markers of astrocytes (GFAP and S100β), neurons (MAP2), microglia (Iba1), and oligodendrocytes (Olig2). The purity of the isolated astrocytes surpassed 99%, as determined by S100β staining quantification (Fig. 1a, b). The contamination of neurons and other glial cells, such as microglia or oligodendrocytes, could be excluded (Fig. 1 and Supplementary Fig. 1). We found that the distribution of glycogen was not homogeneous among astrocytes under transmission electron microscopy. There were few glycogen granules localized in the glycogen^I^ (glycogen-deficient) astrocytes (Fig. 2a). However, glycogen granules were abundantly distributed in glycogen^II^ (glycogen-rich) astrocytes (Fig. 2b). The number of glycogen granules in glycogen^II^ astrocytes was significantly higher than that in glycogen^I^ astrocytes (Fig. 2c).

### Concentration- and Time-Dependent Glycogen Accumulation in Cultured Astrocytes

**Fig. 3:**
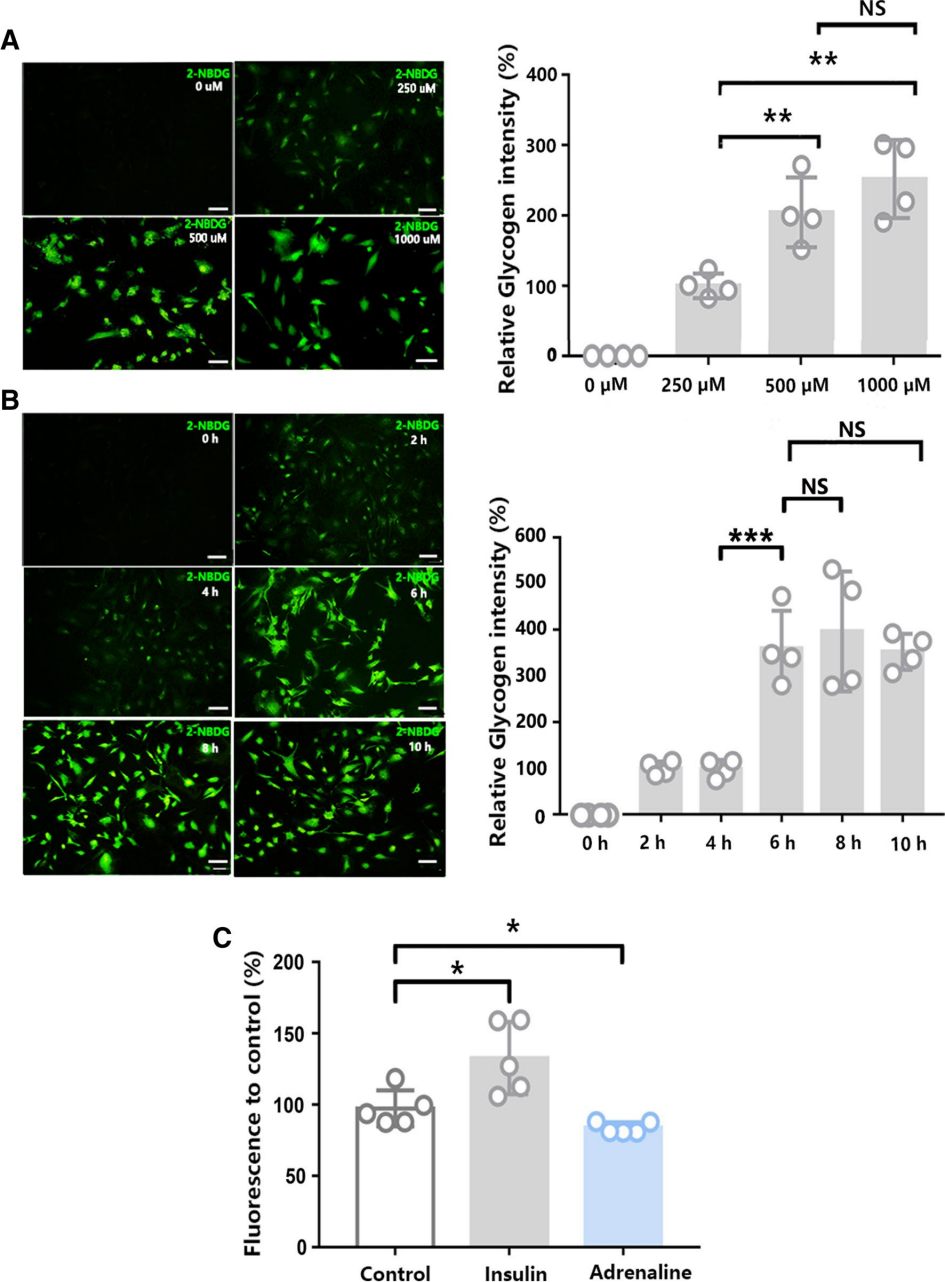
Time and concentration dependence of phosphorylation of 2-NBDG in astrocytes. **a** The fluorescence intensity derived from phosphorylated 2-NBDG in astrocytes increased with the incubation concentration. Fluorescence intensity is presented as the mean ± SEM, and statistical significance was evaluated using one-way ANOVA followed by the Tukey–Kramer post hoc test. *N* = 4 biological replicates. Scale bars = 10 µm. ^**^p < 0.01. **b** Fluorescence intensity derived from phosphorylated 2-NBDG in astrocytes with different incubation times. Fluorescence intensity is presented as the mean ± SEM, and statistical significance was evaluated using one-way ANOVA followed by the Tukey–Kramer post hoc test. *N* = 4 biological replicates. Scale bars = 10 µm. ^***^p < 0.001. **c** Effect of different drugs on fluorescent glycogen. The astrocytes were incubated with 500 µM 2-NBDG for 6 h, and then, nonphosphorylated 2-NBDG was washed out. The results are plotted as the percentage of fluorescence versus control. Samples treated with 5 µM insulin showed significantly higher fluorescence than control samples. Statistical significance was evaluated using one-way ANOVA followed by the Dunnett post hoc test. Adrenaline at 10 µM caused a significant decrease in fluorescence. *N* = 5 biological replicates. ^*^p < 0.05

To determine the diversity of glycogen distribution and metabolism in different astrocytes, we next used 2-NBDG, a deoxyglucose analogue, as a fluorescence maker for glycogen. 2-NBDG could be phosphorylated by hexokinase and taken up as phosphorylated glucose directly by astrocytes (Itoh et al. 2004)*.* Representative images of phosphorylated 2-NBDG in cultured astrocytes are shown in Fig. 3a and b. The cultured astrocytes were incubated with 2-NBDG for either 0, 250, 500, or 1000 µM for 6 h. The nonphosphorylated 2-NBDG (glucose form) was then washed out, and the fluorescence intensity derived from phosphorylated 2-NBDG (glycogen form) was increased in a concentration-dependent manner (Fig. 3a). The fluorescent signals in astrocytes were no longer significantly increased with the increase in 2-NBDG treatment beyond 500 µM. Thus, we chose 500 µM 2-NBDG treatments for the following time-dependent experiments.

Next, we investigated whether phosphorylated 2-NBDG in astrocytes could be time-dependently accumulated. The astrocytes were incubated with 500 µM 2-NBDG for either 2 h, 4 h, 6 h, 8 h, or 10 h, and the nonphosphorylated 2-NBDG was washed out. Fluorescence intensity derived from phosphorylated 2-NBDG increased in a time-dependent manner. The fluorescent signals in astrocytes were no longer significantly increased with the increase in 2-NBDG treatment beyond 6 h (Fig. 3b).

To further confirm that 2-NBDG is a stable marker for astrocytic glycogen content, we used insulin and adrenaline to induce glycogen synthesis or degradation, respectively. Insulin or adrenaline was added to the cell medium after 120 min of incubation with 500 µM 2-NBDG. Then, the cells were further incubated for 30 min with insulin or adrenaline, and the amount of fluorescence retained was detected. The samples were repeatedly washed to remove uncombined 2-NBDG. Insulin caused a rise in 2-NBDG fluorescence intensity. However, adrenaline induced a significant decrease in 2-NBDG fluorescent staining (Fig. 3c). Together, these data indicate that 2-NBDG is a reliable and stable fluorescent tracer for glycogen distribution in cultured astrocytes.

### Glycogen Distribution Patterns in Cultured Astrocytes

**Fig. 4:**
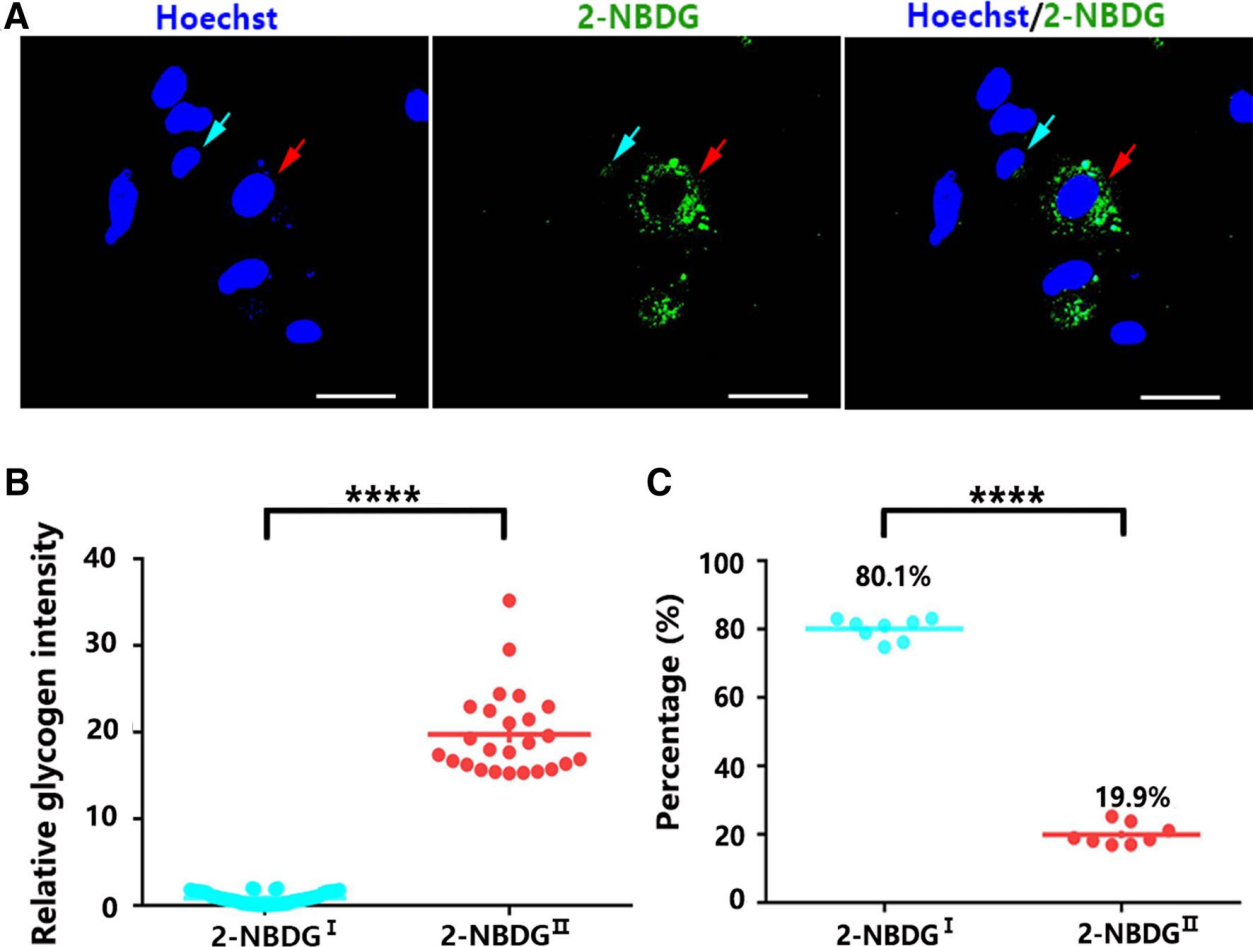
Distribution of glycogen in mouse cortical astrocytes in vitro. **a** The fluorescence intensity from phosphorylated 2-NBDG in cultured astrocytes. Confocal fluorescence showing the fluorescence intensity distribution in astrocytes in vitro. Blue arrows indicate 2-NBDG^I^ cells. Red arrows indicate 2-NBDG^II^ cells. Scale bars = 30 µm. **b** The astrocytes exhibited low and high 2-NBDG fluorescence signals in 2-NBDG^I^ and 2-NBDG^II^. Statistical significance was evaluated using Student’s *t* test. *N* = 124 cells. ^****^p < 0.0001. **c** The results showed that 80.1% of the astrocytes were 2-NBDG^I^ cells, and 19.9% of the astrocytes were 2-NBDG^II^ cells. Statistical significance was evaluated using Student’s *t* test. For each group, 100 to 150 cells were counted. *N* = 8 biological replicates. ^****^p < 0.0001

To further assess the glycogen distribution patterns in cultured astrocytes, we incubated the astrocytes with 500 µM 2-NBDG for 6 h, and green fluorescence derived from phosphorylated 2-NBDG was detected. We found intensive green fluorescence signals in only a small portion of the astrocytes. Most astrocytes had very low fluorescence (Fig. 4a). We separated these astrocytes into two groups: 2-NBDG^I^ (glycogen-deficient) cells with low fluorescence signals and 2-NBDG^II^ (glycogen-rich) cells with high fluorescence signals. The cells with a 2-NBDG fluorescence intensity > 10 were arbitrarily defined as 2-NBDG^II^ astrocytes, while the other cells were defined as 2-NBDG^I^ astrocytes (Fig. 4b). A statistical analysis showed that 19.9% and 80.1% of all astrocytes exhibited high and low 2-NBDG fluorescence signals, respectively (Fig. 4c). We speculated that the astrocytes with different levels of glycogen may have disparate functions.

### Glycogen Metabolic Diversity in 2-NBDG^I^ and 2-NBDG^II^ Astrocytes

**Fig. 5:**
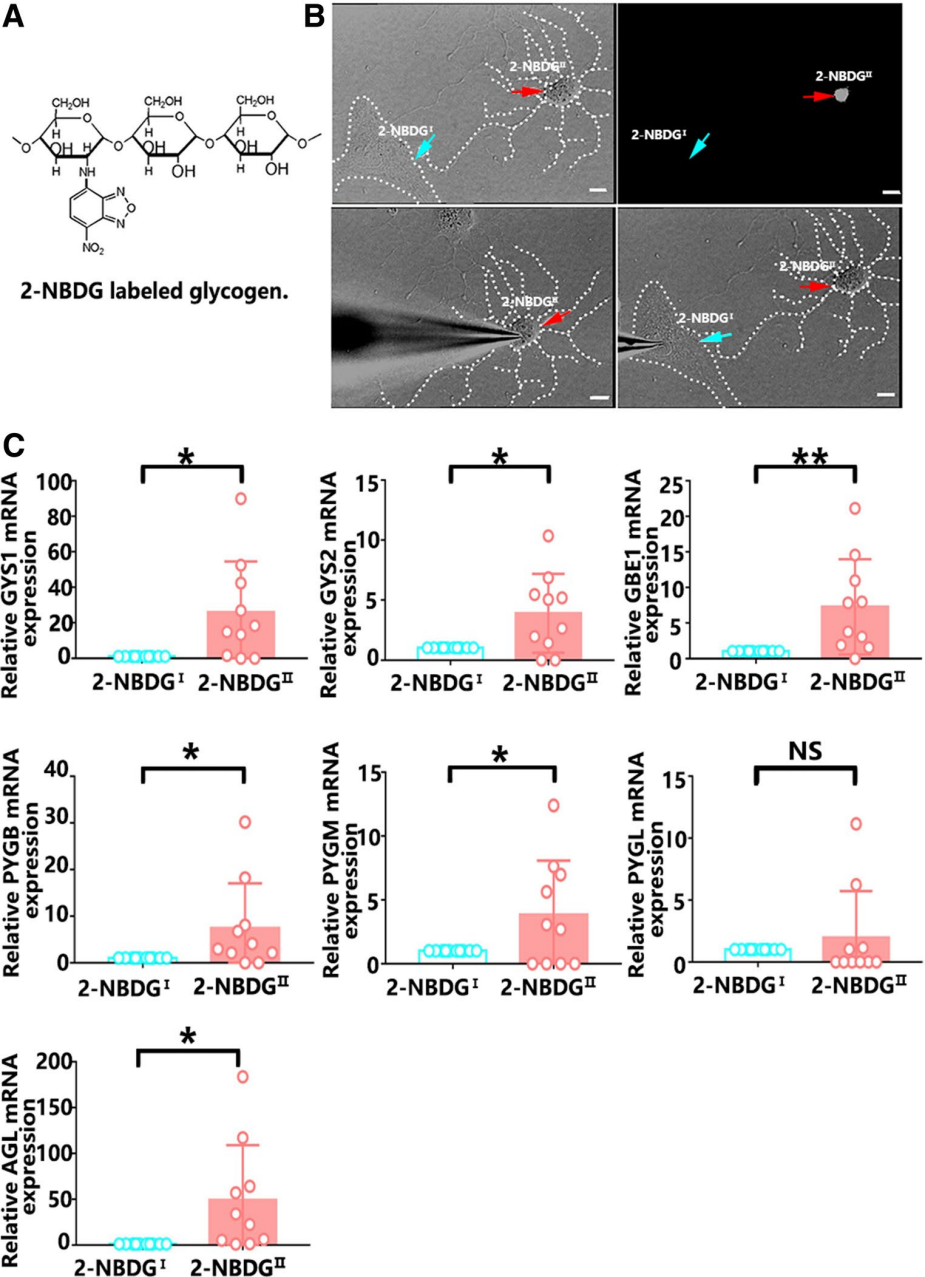
The levels of glycogen metabolism-associated enzymes in single 2-NBDG^I^ and 2-NBDG^II^ astrocytes. **a** The structure of glycogen labeled with 2-NBDG. **b** Patch clamp was used to obtain single cells with different fluorescence intensities. Blue arrows indicate 2-NBDG^I^ cells. Red arrows indicate 2-NBDG^II^ cells. Scale bars = 10 µm. **c** The mRNA levels for glycogen metabolism in 2-NBDG^I^ and 2-NBDG^II^ astrocytes by qRT-PCR using primers specific to the indicated genes: glycogen synthase (GYS1 GYS2), glycogen branching enzyme (GBE1), glycogen phosphorylase brain form (PYGB), glycogen phosphorylase muscle form (PYGM), glycogen phosphorylase liver form (PYGL), and glycogen debranching enzyme (AGL). The statistical significance of gene expression levels was evaluated using Student’s *t* test. *N* = 10 biological replicates. ^*^p < 0.05, ^**^p < 0.01

Glycogen could be bound and labeled by 2-NBDG, resulting in green fluorescence (Fig. 5a). The 2-NBDG^I^ and 2-NBDG^II^ astrocytes showed different levels of glycogen fluorescence staining. To examine the activity of these cells, we performed patch-clamp recording in cultured astrocytes (Fig. 5b). In terms of the resting membrane potential, no significant difference was observed between the 2-NBDG^I^ and 2-NBDG^II^ astrocytes, and these two types of cells were shown to be living cells (Supplementary Fig. 2).

To assess the differences in the glycogen metabolism of the 2-NBDG^I^ and 2-NBDG^II^ astrocytes, we measured the expression levels of seven key enzymes in glycogen synthesis and glycogen breakdown. We used patch clamp to obtain single 2-NBDG^I^ or 2-NBDG^II^ astrocytes (Fig. 5b). Ten pairs of single 2-NBDG^I^ and 2-NBDG^II^ astrocytes were selected to quantify the transcription levels of key enzymes in glycogen metabolism. The mRNA levels of the enzymes in both glycogen synthesis and glycogen breakdown were higher in 2-NBDG^II^ astrocytes than in the 2-NBDG^I^ astrocytes. The qRT-PCR results showed that the expression levels of three key enzymes in glycogen synthesis, GYS1, GYS2 and GBE1, were higher in 2-NBDG^II^ astrocytes than in 2-NBDG^I^ astrocytes. In addition, three key enzymes in glycogen breakdown, PYGB, PYGM, and AGL, had higher expression in 2-NBDG^II^ astrocytes than in 2-NBDG^I^ astrocytes. However, the expression levels of PYGL, another key enzyme in glycogen breakdown, were not significantly different between the two types of astrocytes (Fig. 5c). Together, these results indicate that 2-NBDG^II^ astrocytes show activated metabolism in both glycogen synthesis and glycogen breakdown.

**Fig. 6:**
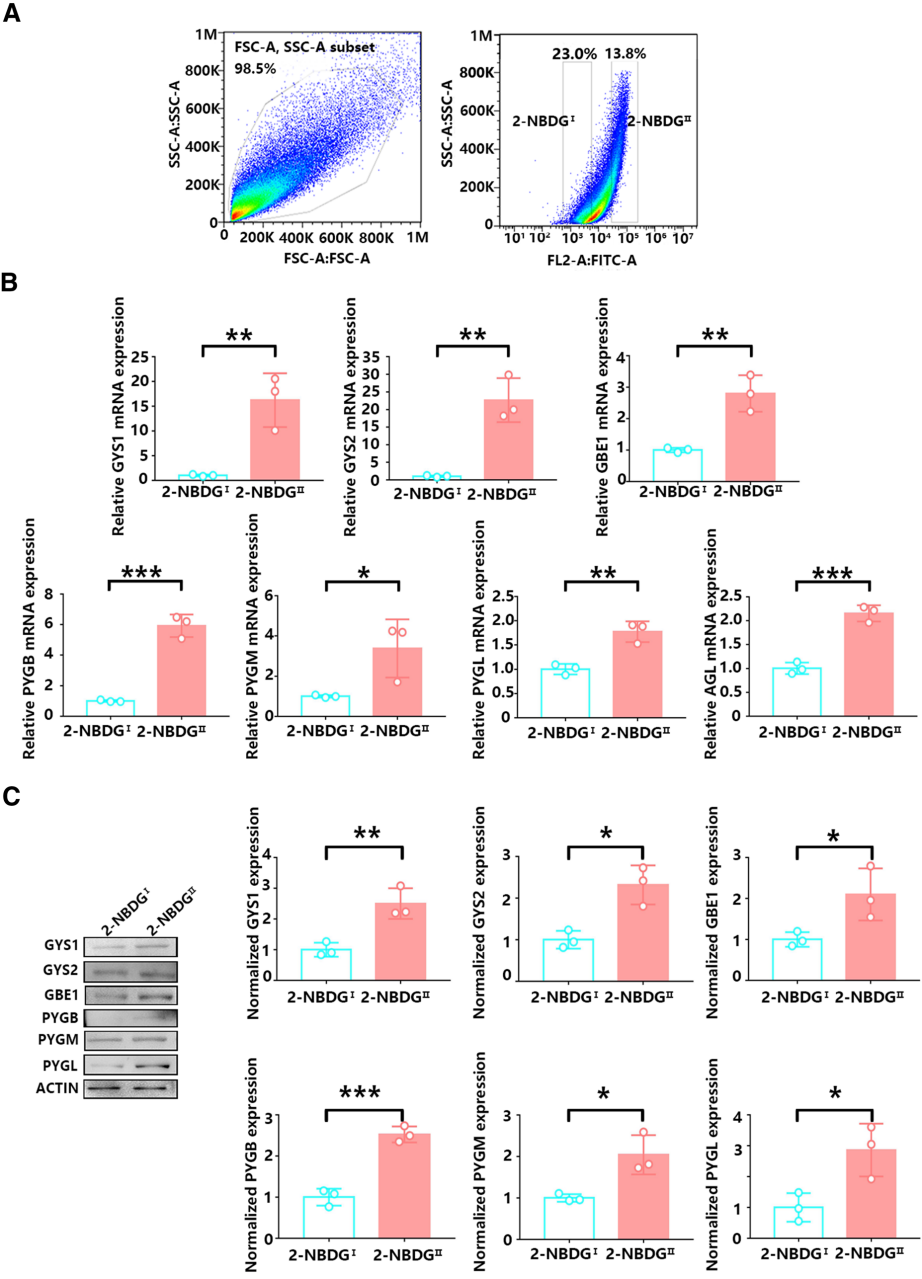
The levels of glycogen metabolism-associated enzymes in two types of astrocytes. **a** Flow cytometric analysis of astrocytes with 2-NBDG revealed different levels of fluorescence. Cells were gated on FITC expression (left panel). The number of gates represents the percentage of astrocytes with fluorescence. The right panel shows different levels of fluorescent expression in astrocytes. The two gates correspond to 2-NBDG^I^ astrocyte and 2-NBDG^II^ astrocyte fluorescence values. **b** The mRNA levels for glycogen metabolism in 2-NBDG^I^ and 2-NBDG^II^ astrocytes by qRT-PCR using primers specific to the indicated genes. The statistical significance of gene expression levels was evaluated using Student’s *t* test. *N* = 3 biological replicates. ^*^p < 0.05, ^**^p < 0.01, ^***^p < 0.001. **c** The protein levels for glycogen metabolism in 2-NBDG^I^ and 2-NBDG^II^ astrocytes by immunoblotting. The statistical significance was evaluated using Student’s *t* test. *N* = 3 biological replicates. ^*^p < 0.05, ^**^p < 0.01, ^***^p < 0.001

Considering the limited number of cells obtained from patch-clamp technology, to determine the protein levels of glycogen metabolism-associated enzymes, we also performed FACS to obtain two groups of astrocytes with high and low fluorescence intensities (Fig. 6a). Consistent with the results of the single cells, FACS analysis showed that the mRNA levels of key enzymes in glycogen synthesis and breakdown were more highly expressed in 2-NBDG^II^ astrocytes than in 2-NBDG^I^ astrocytes (Fig. 6b). Moreover, the protein levels of the key enzymes in 2-NBDG^II^ astrocytes were also substantially higher than those in 2-NBDG^I^astrocytes (Fig. 6c). These findings suggest that 2-NBDG^II^ astrocytes have an enhanced glycogen metabolism compared with 2-NBDG^I^ astrocytes.

### Astrocytic Glycogen Content is Associated with Its Morphology

**Fig. 7:**
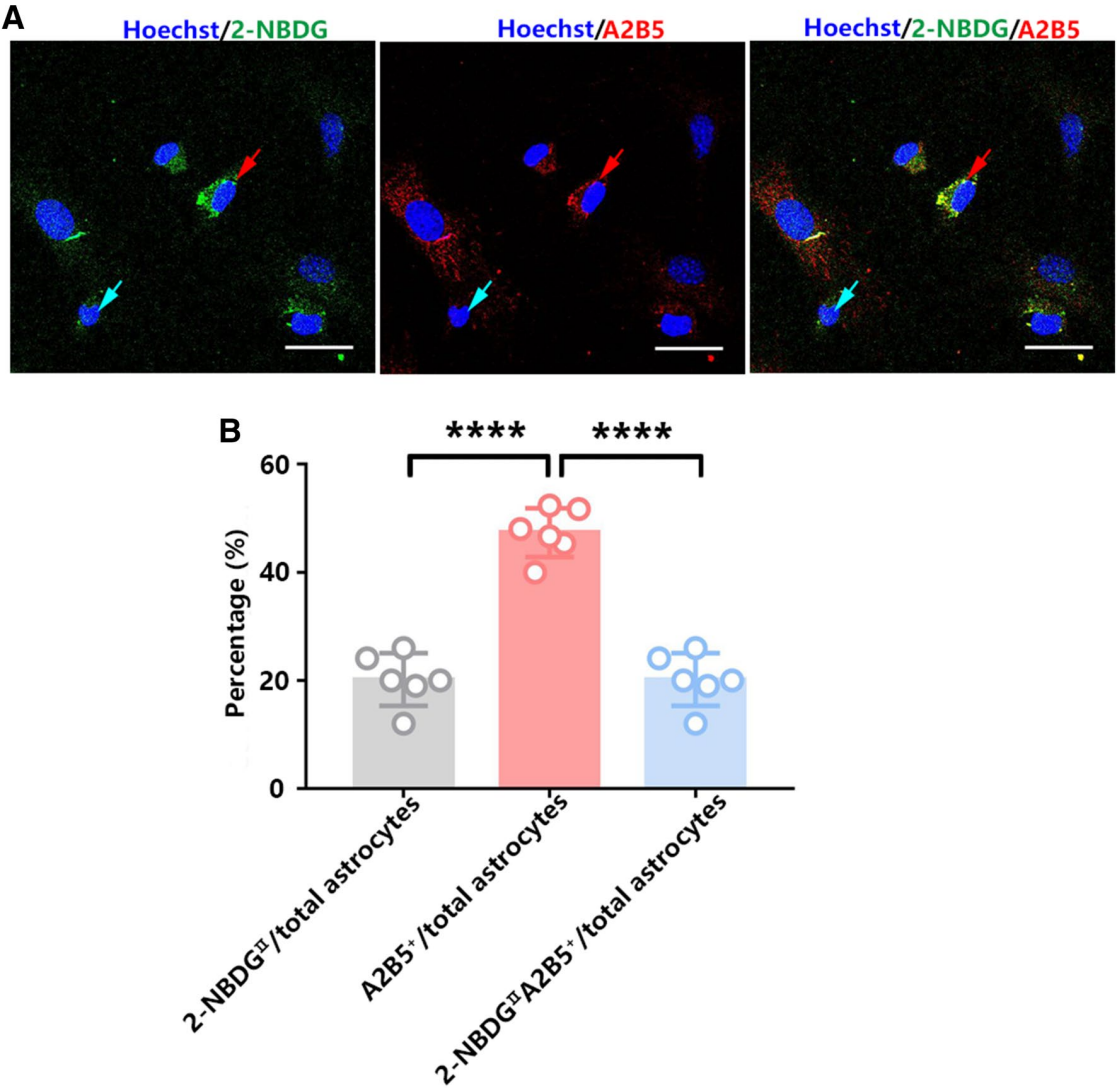
The relationship between astrocytic glycogen and morphology. **a** Representative images of colocalization between phosphorylated 2-NBDG and A2B5 in cultured astrocytes. Blue arrows indicate 2-NBDG^I^ cells. Red arrows indicate 2-NBDG^II^ cell. Scale bars = 30 µm. **b** Percentage of 2-NBDG^II^, A2B5^+^, or 2-NBDG^II^A2B5^+^ cells in total astrocytes. Statistical significance was evaluated using one-way ANOVA followed by the Tukey–Kramer post hoc test. *N* = 6 biological replicates. ^****^p < 0.0001

The differences observed in glycogen metabolism could be a result of the separation of 2-NBDG^I^ and 2-NBDG^II^ cells. Astrocytes have been identified according to their morphologies, protoplasmic and fibrous cells, which can be distinguished with different antibodies. GFAP antibody recognizes both types of astrocytes, while the A2B5 antibody can specifically bind to fibrous cells but not protoplasmic astrocytes (Raff et al. 1984; Bevan and Raff 1985). Here, we found that almost all 2-NBDG^II^ astrocytes were A2B5-positive cells, which accounted for approximately 20% of the total astrocytes, although their colocalization was not complete (Fig. 7a, b). Taken together, the results suggest that brain glycogen primarily localizes in a small portion of fibrous astrocytes.

## Discussion

Here, we showed heterogeneous glycogen distribution patterns in different astrocytic types. Importantly, we found that astrocytes containing higher glycogen have an increased glycogen metabolism, suggesting unique functions of these astrocytes.

Astrocytes can be roughly classified into two types, protoplasmic and fibrous cells. Protoplasmic and fibrous astrocytes are distinct glial cells in antigenic phenotype, developmental history, morphology, and location in the brain (Miller and Raff 1984). Here, we first identified that these two types of cells also differ in the amount of glycogen. As shown by the electron microscopy results, abundant glycogen localizes in type II astrocytes but not in type I astrocytes. However, the relationship between cell morphology and the two types of astrocytes is unknown.

Here, we used a fluorescently labeled d-glucose, 2-NBDG, to label brain glycogen. We found that phosphorylated 2-NBDG fluorescence was principally distributed in a small portion of astrocytes rather than all astrocytes. Therefore, we sought to clarify whether there are any differences in glycogen metabolism between 2-NBDG^I^ (glycogen-deficient) and 2-NBDG^II^ (glycogen-rich) astrocytes.

The patch-clamp technique was used to obtain single cells with different fluorescence intensities. No significant difference in the resting membrane potential was observed between 2-NBDG^I^ and 2-NBDG^II^ astrocytes, and they were all living cells. The FACS technique was used to verify the levels of glycogen metabolism in 2-NBDG^I^ and 2-NBDG^II^ cells. Most key enzymes in glycogen synthesis and catabolism were upregulated in 2-NBDG^II^ cells compared with 2-NBDG^I^ cells. Our results indicated that 2-NBDG^II^ astrocytes have more vigorous glycogen metabolism than 2-NBDG^I^ astrocytes, suggesting functional diversity associated with energy metabolism for different astrocytes.

A2B5 is a specific cell marker for fibrous astrocytes. We found colocalization between A2B5 and the 2-NBDG fluorescent signal, although the colocalization was not complete. Together, the data from the A2B5 and 2-NBDG fluorescent signals indicated that brain glycogen principally exists in the cytoplasm of fibrous astrocytes but not protoplasmic astrocytes. We speculated that the two types of astrocytes with different glycogen metabolism are involved in different neural functions. Analysis of the spatial relationship among specific astrocytes, neurons, and microvessels may provide clues regarding the complicated and elaborated functional mechanisms for heterogeneous astrocytic glycogen localization in the future.

In summary, we identified distinct glycogen localization and glycogen metabolism between two types of astrocytes, suggesting different functions of these heterogeneous cells that are related to energy requirements.

## Electronic supplementary material

Below is the link to the electronic supplementary material.

Supplementary file1 (DOCX 980 kb)
