# Supplementary material for: Single-Cell Analysis for Glycogen Localization and Metabolism in Cultured Astrocytes
Source: Cell Mol Neurobiol. 2019 Dec 20;40(5):801–12. doi: 10.1007/s10571-019-00775-4 (PMC7261284; doi:10.1007/s10571-019-00775-4)
Supplement: Supplementary file 1 — Supplementary file1 (DOCX 980 kb) [file 10571_2019_775_MOESM1_ESM.docx]

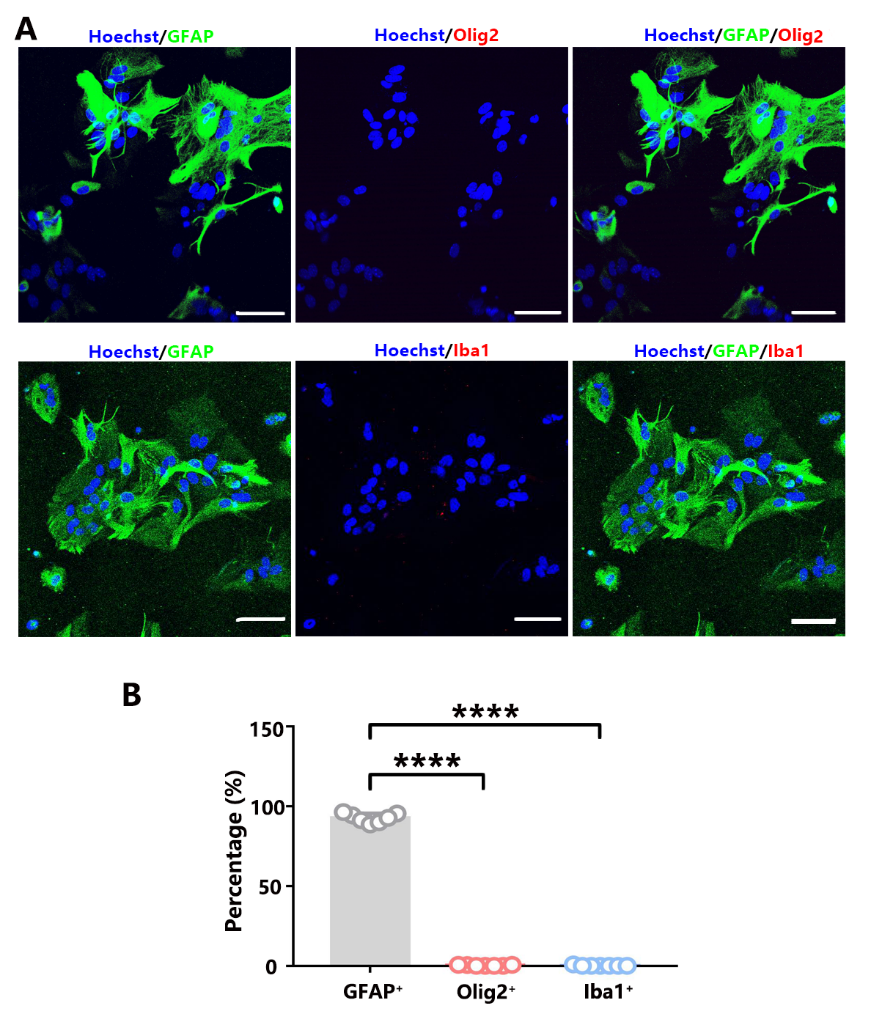


**Supplementary Figure 1** The purity of the astrocytes was confirmed by immunofluorescence. **(A)** Costaining with GFAP and Olig2 or Iba1 in cultured astrocytes. Scale bars = 30 µm. **(B)** Quantification of GFAP-, Olig2-, and Iba1-positive cells. Statistical significance was evaluated using one-way ANOVA followed by the Tukey-Kramer post hoc test. N = 7 biological replicates. ^****^*p* < 0.0001.


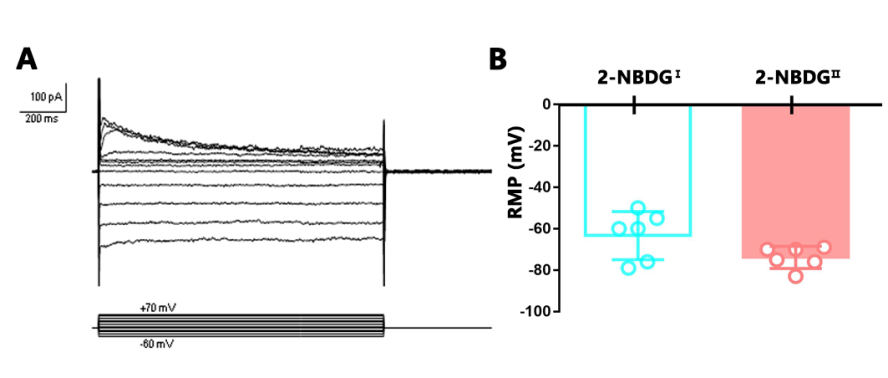


**Supplementary Figure 2** Electrophysiological properties of 2-NBDG^Ⅰ^ or 2-NBDG^Ⅱ^ astrocytes. **(A)** Cells were given electrical stimulation (1 s) from -60 to 70 mV with 10 mV steps. The cells generated potassium currents but no sodium currents. **(B)** Resting membrane potentials (RMP) of the 2-NBDG^Ⅰ^ or 2-NBDG^Ⅱ^ astrocytes. Statistical significance was evaluated using Student’s t test, N = 6 biological replicates.
